# Concerns among people who use opioids during the COVID-19 pandemic: a natural language processing analysis of social media posts

**DOI:** 10.1186/s13011-022-00442-w

**Published:** 2022-03-05

**Authors:** Abeed Sarker, Nisha Nataraj, Wesley Siu, Sabrina Li, Christopher M. Jones, Steven A. Sumner

**Affiliations:** 1 Department of Biomedical Informatics, School of Medicine, Emory University, GA 30322 Atlanta, Georgia; 2 National Center for Injury Prevention and Control, Centers for Disease Control and Prevention, GA 30341 Atlanta, Georgia; 3 Rollins School of Public Health, Emory University, GA 30322 Atlanta, Georgia; 4 Department of Computer Science, Emory University, GA 30322 Atlanta, Georgia

**Keywords:** Opioids (MeSH ID: D000701), Opioid use disorder (MeSH ID: D009293), COVID-19 (MeSH ID: D000086382), Coronavirus (MeSH ID: D017934), Natural language processing (MeSH ID: D009323), Text mining (MeSH ID: D057225), Social media (MeSH ID: D061108)

## Abstract

**Background:**

Timely data from official sources regarding the impact of the COVID-19 pandemic on people who use prescription and illegal opioids is lacking. We conducted a large-scale, natural language processing (NLP) analysis of conversations on opioid-related drug forums to better understand concerns among people who use opioids.

**Methods:**

In this retrospective observational study, we analyzed posts from 14 opioid-related forums on the social network Reddit. We applied NLP to identify frequently mentioned substances and phrases, and grouped the phrases manually based on their contents into three broad *key* themes: (i) *prescription and/or illegal opioid use*; (ii) *substance use disorder treatment access and care*; and (iii) *withdrawal*. Phrases that were unmappable to any particular theme were discarded. We computed the frequencies of substance and theme mentions, and quantified their volumes over time. We compared changes in post volumes by key themes and substances between pre-COVID-19 (1/1/2019—2/29/2020) and COVID-19 (3/1/2020—11/30/2020) periods.

**Results:**

Seventy-seven thousand six hundred fifty-two and 119,168 posts were collected for the pre-COVID-19 and COVID-19 periods, respectively. By theme, posts about treatment and access to care increased by 300%, from 0.631 to 2.526 per 1000 posts between the pre-COVID-19 and COVID-19 periods. Conversations about withdrawal increased by 812% between the same periods (0.026 to 0.235 per 1,000 posts). Posts about drug use did not increase (0.219 to 0.218 per 1,000 posts). By substance, among medications for opioid use disorder, methadone had the largest increase in conversations (20.751 to 56.313 per 1,000 posts; 171.4% increase). Among other medications, posts about diphenhydramine exhibited the largest increase (0.341 to 0.927 per 1,000 posts; 171.8% increase).

**Conclusions:**

Conversations on opioid-related forums among people who use opioids revealed increased concerns about treatment and access to care along with withdrawal following the emergence of COVID-19. Greater attention to social media data may help inform timely responses to the needs of people who use opioids during COVID-19.

**Supplementary Information:**

The online version contains supplementary material available at 10.1186/s13011-022-00442-w.

## Background

As of June 2021, the novel coronavirus disease (COVID-19) pandemic has claimed the lives of over 590,000 individuals in the US and 3.8 million globally [1]. Public health experts suggest that the intersection of the COVID-19 pandemic and the ongoing drug overdose epidemic has had detrimental impacts on efforts to reduce deaths [2, 3]. Estimates in the US show that monthly increases in overdose deaths during April and May 2020, the early months of the pandemic, were the largest since provisional data reporting began in 2015 [4].

Persons with substance use disorder (SUD) are thought to be directly at a higher risk for COVID-19 and poor outcomes because of higher rates of immunocompromised status and other underlying comorbidities [3]. Indirectly, disruptions in access to SUD treatment and mental health services have made it more difficult for patients to receive timely and consistent care [5], and social distancing measures have resulted in individuals using substances alone, raising risk for overdose death [6, 7]. There are additional concerns that housing, employment, and economic instability, correlates of substance use problems, have substantially increased during the pandemic [8, 9].

However, little is known about immediate concerns among communities of people who use prescription and illegal opioids, and how those concerns have evolved during the COVID-19 pandemic. Our goal was to harness large-scale, publicly-available conversation data from posts made on Reddit, which houses the largest online opioid-related drug forums, to better understand how communities of people who use opioids have been impacted by the pandemic. We use natural language processing (NLP) and a mixed-methods design to study trends in topics prior to and since the emergence of the COVID-19 pandemic. Improved, timely awareness of issues affecting people who use opioids during the pandemic can help local, state, and federal organizations better respond to the needs of these communities in the moment.

## Methods

### Data source

Reddit, one of the largest social networks and the largest online forum with over 52 million daily users, hosts active community forums on a range of substance-use related topics including, but not limited to, opioid use and has been extensively used for similar research [10]. We identified 14 forums (i.e., “subreddits”) containing discussions on prescription and illegal opioid use and recovery. We retrieved historical public post data using the Python-Reddit Application Programming Interface Wrapper.^1^ The Emory Institutional Review Board determined this secondary research using publicly available information exempt from review. We divided historical posts in the subreddits into two data subsets to study any changes in conversation following the emergence of the pandemic:

- *pre-COVID-19 period*: January 1, 2019–February 29, 2020.
- *COVID-19 period*: March 1, 2020–November 30, 2020.

### Data processing

The included subreddits span topics ranging from opioid use disorder (OUD) recovery strategies to personal experiences with opioids. Consistent with standard convention in NLP research, posts were initially preprocessed by converting text to lowercase, reducing words to their stems (i.e., stemming), and removing stopwords (e.g., ‘a’, ‘the’, ‘in’, etc.) using the Python Natural Language Toolkit.^2^

Next, potential phrases of interest were identified quantitatively by calculating:

- Frequency distributions of words/phrases 1–3 tokens long for each study period.
- Term frequency inverse document frequency (TF-IDF) values, including monthly and aggregate TF-IDF values for both periods. TF-IDF calculations assign weights to a given word/phrase by frequency of appearance in a data subset relative to posts from the remaining dataset. We employed a customized TF-IDF method where “TF” denotes the term frequency for a given period (i.e., month) and “IDF” denotes “the inverse of the term frequency” from other periods (Supplementary material A).

Subject matter experts in substance use (CMJ and SAS) then manually reviewed the phrases of interest, combined with their spelling and phrasing variants, to identify the top 50 unique terms relevant to substance use (Supplementary material B). These were then used to explore two main questions: how did the emergence of COVID-19 shift 1) substance-use related themes being discussed? and 2) conversations around specific substances?

### Shifts in substance use related themes

The subject matter experts grouped the selected terms into three broad themes by primary content discussed: 1) prescription and illegal opioid use, 2) SUD treatment and access to care, and 3) withdrawal. Relative frequencies were then calculated by month for each theme using the total monthly number of posts from the 14 included subreddits as the denominator. We also plotted trends in the frequency of the term ‘COVID’. Since this term was understandably absent before the pandemic, its rise following the outbreak served as a suitable comparison for other substance-related themes explored. Qualitative review of posts by theme was conducted to validate our automatic findings and further explore the nature of conversations.

### Shifts in specific drug mentions over time

We also studied changes in the mentions of specific substances in the two periods. Drugs of interest were identified using the aforementioned quantitative and qualitative approaches (Appendices-C-D). We combined brand, generic, and street names for substances into single items, where necessary. Since drug names are often misspelled or expressed using non-standard expressions on social media, we used an automatic variant generator to include commonly-used lexical variants. We then computed the relative frequencies of these substances in the pre-COVID-19 and COVID-19 periods to explore trends. All analyses were conducted in Python (Version 3.8).

## Results

**Fig. 1:**
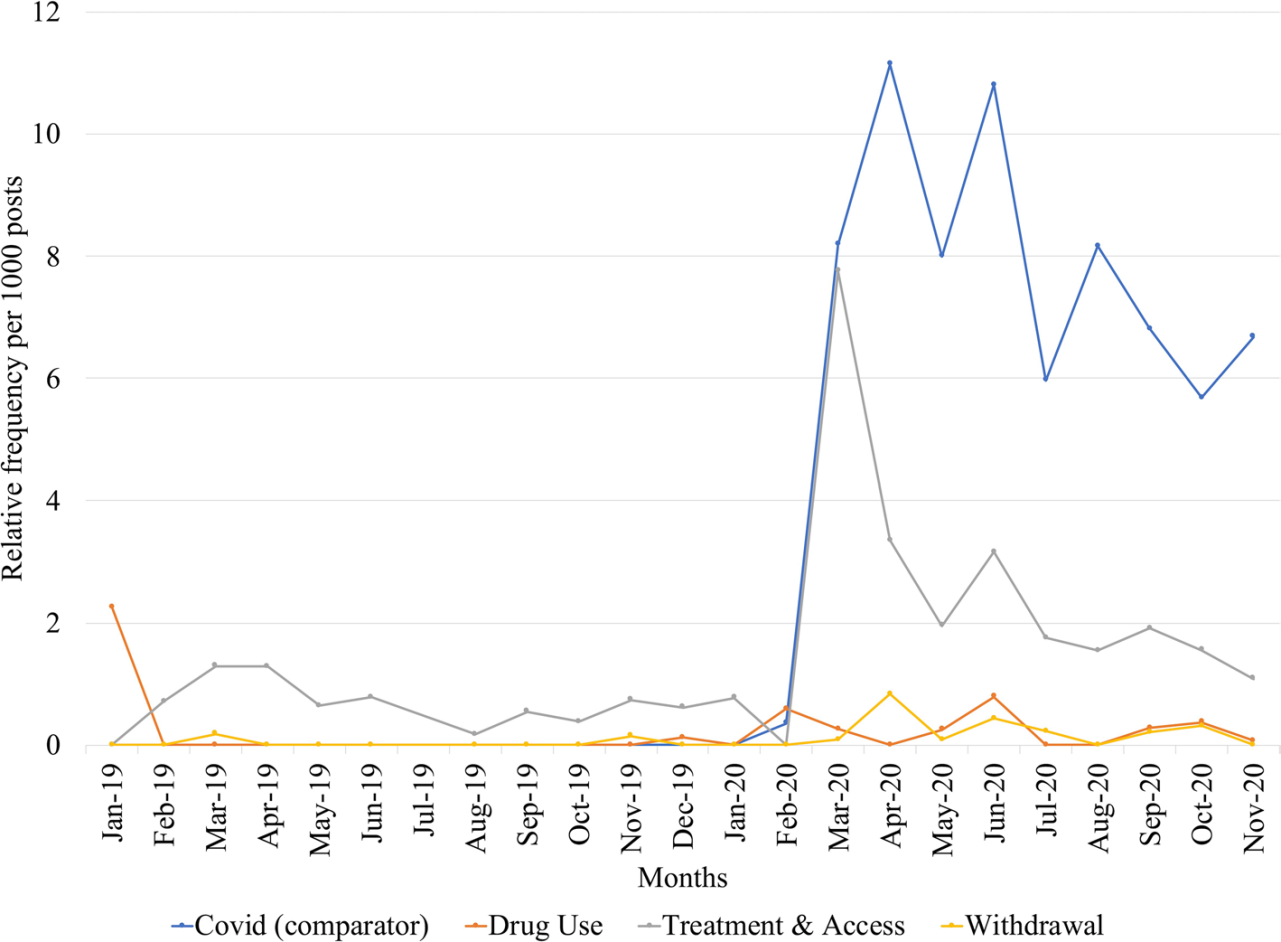
Month-to-month relative frequencies for expressions belonging to the themes (i) drug use, (ii) treatment and access, and (iii) withdrawal. Relative frequencies for the term 'covid' are provided for comparison

Posts were collected and analyzed across the 14 opioid-related subreddits, with 77,652 and 119,168 posts during the pre-COVID-19 and COVID-19 periods, respectively. Figure 1 shows the monthly relative frequencies of the major themes of drug use, treatment and access, and withdrawal per 1,000 posts. For comparison, “COVID” was rarely mentioned before March 2020, but its relative frequency rapidly rose once the pandemic received widespread attention. Of all themes, there was a considerable increase in posts about treatment and access to care following the emergence of the pandemic which increased 300% from 0.631 in the pre-COVID-19 period to 2.526 per 1,000 posts. Posts concerning this theme were most prevalent during March 2020, accounting for approximately 8 out of 1,000 posts. Conversations about withdrawal also increased in the COVID-19 period, albeit smaller in scale compared to treatment and access conversations, exhibiting an 812% rise from 0.026 to 0.235 per 1,000 posts. Posts about drug use did not increase during the COVID-19 period (0.219 to 0.218 per 1000 posts; -0.001% change).

Table 1 presents an examination of changes in the average daily frequency of specific substances mentioned. There was a consistent increase in mentions of opioids and certain other substances. Methadone, a medication for opioid use disorder, demonstrated the largest increase (20.751 to 56.313; 171.4%). Among other medications, diphenhydramine experienced the largest relative increase in mentions on opioid-related forums, followed by benzodiazepines. Kratom and Iboga, other substances not used in medical settings, also had increases.

**Table 1:** Change in post-volume pre-COVID-19 and COVID-19 periods among leading substances discussed on opioid-related forums

	**Pre-COVID-19 period daily average**	**COVID-19 period daily average**	**Difference**	**Change** (%)
**Medications for Opioid Use Disorder and Overdose Reversal**				
Methadone	20.751	56.313	35.562	171.4%
Naltrexone	1.087	2.451	1.364	125.5%
Buprenorphine	18.426	36.225	17.800	96.6%
Naloxone	1.680	3.116	1.436	85.5%
**Other Substances used in Non-Medical Settings**				
Iboga	0.520	1.175	0.655	125.9%
Kratom	3.454	7.222	3.768	109.1%
**Prescription and Illegal Opioids**				
Hydrocodone	0.986	2.687	1.701	172.6%
Fentanyl	9.506	25.404	15.898	167.2%
Oxycodone	2.191	5.796	3.606	164.6%
Other prescription opioids	1.605	3.687	2.083	129.8%
Heroin	8.325	14.677	6.352	76.3%
Carfentanil	0.325	0.411	0.086	26.5%
**Other Medications**				
Diphenhydramine^a^	0.341	0.927	0.586	171.8%
Diazepam	0.504	1.324	0.820	162.9%
Other benzodiazepines	4.791	11.178	6.388	133.3%
Alprazolam	1.384	3.164	1.780	128.7%
Dextromethorphan^a^	0.146	0.291	0.145	99.4%
Dextroamphetamine	1.252	2.131	0.879	70.2%

Substances are ordered by percentage change within each medication grouping. Category of “other opioids” and “other benzodiazepines” represents all such medications not explicitly mentioned in the table. Counts are based on generic and trade names, and their common misspellings

^a^Denotes substances available in over-the-counter forms

Qualitative assessment of posts that contained treatment and access to care related content revealed that the majority of posts were from individuals currently in recovery. A large volume of conversations focused on take-home access to methadone. For example, one post (abbreviated for clarity and anonymity) stated: … “*The most [take-homes] one can get at [my opioid treatment program] is 1 week. I asked if they would have to make special exceptions because of the [COVID-19] crisis. I was told that nobody has to do anything for us… I understand that take home medications could kill kids, pets, *etc.*[.] if someone is irresponsible[,] but I think they should let up a little bit.”.* Other posts highlighted changes opioid treatment programs had made since the onset of the pandemic: “… *I usually have 2 week [take-homes], I'm getting 28 days now [because] of COVID19. My clinic has ONLY been testing unstable patients, new patients, or ones with recently failed [urinalyses]. But all of it is up to the individual clinics or corporate owners of the clinic.*”. Additional examples are available in Supplementary material E.

## Discussion

This study used large-scale real-world conversation data to explore the concerns of people who use opioids after the emergence of the COVID-19 pandemic. Our mixed-methods approach reveals that conversations about treatment and access to care increased markedly during COVID-19, with a focus on methadone access and increased discussion of over-the-counter medications (e.g., diphenhydramine) and other substances (e.g., kratom).

There is increased demand for timely data to guide public health prevention activities as health professionals have raised several potential concerns facing SUD treatment and overdose prevention arising from the COVID-19 pandemic [11, 12]**.** Leading sources of information on drug use traditionally include large-scale surveys such as the National Survey on Drug Use and Health and data derived from toxicology testing of overdose deaths [13]. Unfortunately, due to time-consuming nature of these data collection methodologies, real-time data from these official sources are not available to guide decision making. Consistent with other studies [14, 15], our results highlight the role and relevance of novel data sources to explore early insights and complement traditional data systems.

Manual examination of posts verified that the NLP analysis findings are indeed informative. We found large increases in conversations about treatment and access to care from March to November 2020. For methadone, a considerable amount of the increase concerned regional policies about medications for opioid use disorder access. In March 2020, the Substance Abuse and Mental Health Services Administration released guidance allowing opioid treatment programs to provide take-home doses of methadone and buprenorphine for established, stable patients. Under access to care, discussions surrounding methadone increased the most relative to other medications for opioid use disorders. Interestingly, while policy changes attempted to facilitate access to medications for opioid use disorder during COVID-19, access to methadone was under stricter regulation than buprenorphine; for example, clinicians at opioid treatment programs could initiate buprenorphine remotely for new patients without an in-person examination unlike methadone initiation which still required in-person examinations. Additionally, in office-based settings, clinicians with a Drug Addiction Treatment Act waiver to prescribe buprenorphine for OUD were provided emergency authority to initiate new patients remotely without in-person examinations.

Despite considerable speculation about how COVID-19 is shifting substance-use markets and patterns, we found that conversations among people who use opioids were overwhelmingly focused on treatment relative to conversations about procuring and using illegal substances. This underscores the interest in medications for opioid use disorder, despite the myriad challenges that face the medications for opioid use disorder maintenance, especially during the pandemic. We note that our analysis did reveal an increase in conversations about fentanyl during the COVID-19 period when overdose deaths involving synthetic opioids such as illegally made fentanyl accelerated [16].

Non-opioid substances showed marked increases in conversations within the opioid subreddits, the largest of which were seen for over-the-counter (e.g., diphenhydramine) or prescription (e.g., benzodiazepines) medications used to treat symptoms, including those associated with withdrawal [17]. Of note, benzodiazepines have been known to be co-ingested with opioids, often with fatal consequences [18]. Kratom, which is unregulated, and iboga (ibogaine), a schedule-I controlled substance also showed increases in conversations. These substances have been of interest to people who use opioids as alternatives for opioid treatment or withdrawal in non-medical settings and have received widespread social media attention [19] despite concerns about safety [20, 21]. Insights from our work may help inform population-level monitoring efforts for drug poisoning and overdose education by providers and public health practitioners, specifically in the context of COVID-19.

This work has some limitations. Although the data sources employed represent large-scale data from the largest drug forums online, we recognize that such data are a convenience sample and may not fully represent concerns of populations who do not use these platforms. Additionally, stay-at-home orders could have resulted in conversations about the studied themes shifting from in-person to online channels. Thus, results from these efforts should be used to help generate early hypotheses and insights that can be further assessed using traditional health and substance use surveillance systems. Furthermore, this study uses qualitative review of posts; future work should evaluate the accuracy of fully-automated, unsupervised machine learning methods to further accelerate the timeliness of analyses and insights.

Nevertheless, our findings show that treatment and access to care continue to be major themes discussed by people who used opioids during the COVID-19 pandemic. Rapidly identifying and addressing concerns of these communities through novel data and analytic methodologies are crucial. Removing barriers to care, including through the expansion of telehealth and allowance of take-home doses of medications for opioid use disorder from opioid treatment programs, are important to support overdose prevention activities and long-term recovery as part of the overdose public health emergency response.

## Conclusion

Our analysis demonstrates the utility of social media for studying SUD, particularly at times when significant social changes occur, such as the spread of the COVID-19 pandemic. There is still considerable uncertainty about how COVID-19 will keep impacting the lives of people. COVID-19 resulted in substantial increases in chatter related to treatment and access, and withdrawal. We found that this was because people suffering from SUD had major concerns about maintaining their treatment once stay-at-home orders were issued. Active surveillance using social media data can help track emerging concerns and problems as the world prepares for post-COVID-19 life. Currently, despite the usefulness of social media for SUD research and surveillance, they have been largely under-utilized. We believe that caregivers and policy makers should put greater attention to social media data, as they may help inform timely responses to the needs of people who use opioids during COVID-19.

## Supplementary Information

**Additional file 1.** A.TF-IDF equation. B.Top 50 selected terms and phrases. C. Drug names and misspellings. D. Medication names and grouping. E. Sample posts.

## Data Availability

Publicly available data from Reddit was used for this study. Authors do not have the authority to release data beyond the examples in the manuscript and supplementary material. Data can be obtained directly from Reddit via the appropriate application programming interface (API).

## Abbreviations

OUD: Opioid use disorder
SUD: Substance use disorder
NLP: Natural language processing
TF-IDF: Term frequency—inverse document frequency

## Footnotes

1 Boe B. PRAW: The Python Reddit API Wrapper. (https://praw.readthedocs.io/en/latest/). Accessed: 06/21/2021.

2 https://www.nltk.org/. Accessed: 06/21/2021.
