# Supplementary material for: Concerns among people who use opioids during the COVID-19 pandemic: a natural language processing analysis of social media posts
Source: Subst Abuse Treat Prev Policy. 2022 Mar 5;17:16. doi: 10.1186/s13011-022-00442-w (PMC8897722; doi:10.1186/s13011-022-00442-w)
Supplement: Supplementary file 1 — Additional file 1. A.TF-IDF equation. B.Top 50 selected terms and phrases. C. Drug names and misspellings. D. Medication names and grouping. E. Sample posts. [file 13011_2022_442_MOESM1_ESM.docx]

Supplementary material

Title: Impact of the COVID-19 Pandemic on People who use Opioids: A Natural Language Processing Analysis of Social Media Posts

1. TF-IDF equation

$${tf-idf}_{modified}=term freq. this time period \times\frac{1}{term freq. other time periods}$$

2. Top 50 selected terms and phrases and their categories

| Treatment and access | Withdrawal | Drug Use |
| --- | --- | --- |
| find new doc | acut withdraw stage | harder get high |
| take home tomorrow | mild withdraw | take heroin |
| take home everyon | withdraw tri make | mexican oxi |
| covid take home | withdraw becom | cotton fever |
| extend take home | clonidin help lot |  |
| give week take | get past day |  |
| normal take home | nervou stomach |  |
| take home covid | detox long |  |
| clinic give takehom | unstabl patient |  |
| coupl day take | feel like crap |  |
| give takehom peopl | wors bodi |  |
| peopl get week | atm withdraw |  |
| take home miss |  |  |
| take home pandem |  |  |
| extra takehom |  |  |
| everyon takehom |  |  |
| take home tomorrow |  |  |
| give extra take |  |  |
| given extra |  |  |
| take home |  |  |
| methadone clinic |  |  |
| clinic stay open |  |  |
| swapped |  |  |
| brand sub |  |  |
| sub bc |  |  |
| bring lock box |  |  |
| clinic stay open |  |  |
| usual get week |  |  |
| clinic give extra |  |  |
| fill earli |  |  |
| offer money |  |  |
| blue methadon |  |  |
| bottl left |  |  |
| pharmaci refus |  |  |
| *Terms used for trend comparison, COVID-19: covid, coronavirus, corona virus, covid19 | | |

1. **Drug names and misspellings:**
2. Fentanyl: fentinyl fentenyl fenanyl fentanly fentnyal fentanol fental fetanyl fentayl fentanayl fentanyl fentyl fentanal fetnyl fentynyl fentnayl fentanl fentyanl fentonyl fentanyal fentany fentnyl
3. Carfentanyl: carfentanyl
4. Suboxone: suboxine subuxone suboxne suboxone saboxone seboxone subxone suboxene suboxones soboxone suboxon
5. Sublocade sublocade
6. Methadone: methodone methadon methadrone methadose methadones methadone mathadone methedone metadone mehtadone methdone
7. Blue_methadone: blue_methadone
8. Dextromethorphan: dextromethorphan dextromethorphan_hbr dextromorphan dxm_dextromethorphan dextromethorphan_dxm
9. Phenylephrine: phenylephrine_hcl phenylephrine
10. Buprenorphine: bupenorphine burprenorphine bupronorphine buprenophine bupernorphine buprenorphin buprenorphine
11. Expectorants: expectorant expectorants
12. Chlorpheniramine: chlorpheniramine
13. Dimenhydrinate: dimenhydrinate
14. Diphenhydramine: diphenhydramine diphenhydramine_hci diphenhydramine_50mg dipenhydramine dyphenhydromine diphenhydramine_hcl diphenhydromine dyphenhydramine
15. Brompheniramine: brompheniramine
16. Cough suppressant: cough_suppressants cough_suppressant
17. Hydrocodone: hyrdrocodone hydrocodiene hydro_codone hydrocodene hydrocordone hydorcodone hydrocodeine hidrocodone hyrocodone hydrocone hydrodone hyrdocodone hydrcodone hydrocodones hydrocordisone hydocodone hydrocode hydrocodons hydracodone hydrocodone hydrocodin hydrocodine hydrocodon hydrocondone hydrocodne hydrocdone
18. Vicodin: vicoden vicadin viodin vicodin vicodines vicodan vicodien viccodin vocodin vicondin vicoding vicodins vicodon vicidin vidodin vikodin viacodin vicodine vicdin vicotin
19. Dxm: dxm
20. Benadryl: benadyl benedryl beadryl benadryls benidryl benadrly benadrl benadril bynadryl benadryl bendadryl benadryll bendaryl bennadryl bendryl bendryal benadryal benadry benydryl benadryle bendryle
21. Percocet: perkocet percocete percacet pecocet percocette perocets percoets percoet percot perocet percoset percocets percocett pecocets percocoet percocit percet percoct percocet10 percicet percocetes percecet percocet
22. Oxycodone: oxocodone oxycodene oxycondone oycodone oxyxodone oxycodones oxicodone oxy_codone oxycodone oxycodine roxycodone ocycodone oxycodons oxcodone oycondone oxycodon oyxcodone oxcycodone oxycodone_💊💊 oxycodne oxycodone_💊
23. Oxycontin: ocycontin oxcontin oxcotin oxcycontin oxycotine oxycontin roxycontin oycotin oxyconton oxycontine oxycotins oxycotin oxycintin oxy_contin oxicontin oxycontins oxycottin oycontin
24. Benzo: benzos benzo benzio
25. Sleep aid: sleep_aid
26. Xanax: xanax xannax xanax😂 xanaxs xnax xanx xzanax xanxax xananx xnanax axanax xanaxx xantax xanaax
27. Valium: valium vallium valliums valim vaium valerium valum valiums valiium
28. Ativan: attivan ativan antivan ativans atvan activan
29. Benzodiazepine: benzodiazopine benzodiazipines benzodiazepene benzodiazepans benzodiazepines benzodiazepine benzodiazapene benzodiazipine benzodiazapines benzodiazepenes benzodiazpines benzodiapines benzodiazopines benzodiazapine benzodiazapins
30. Alprazolam: loprazolam alprazalam alprozolam alpraxolam alprazolam aprazolam
31. Adderall: adderall addrerall sadderall adderell addrall adderals addera adderall adderoll addorall addarall adderrall dadderall adderall_📲 aderal adderall_🆘 adderalll adderallll adderal badderall smadderall adderall_💊 addreall adderallxr madderall adderall_🔌 adderall🎶 aderall aderrall adderral adderally adderalls seadderall
32. Kratom: kratom
33. Sleep med: sleep_med
34. Naloxone: naloxone nalaxone
35. Naltrexone: naltrexone naltraxone natrexone naltrexon naltroxone
36. Vivitrol: vivitrol
37. Ibogaine: ibogain ibogaine
38. Narcan: narcan
39. Heroin: heroin herroin herioin heroins
40. Medication names and grouping

| **Medication** | **Category** | **Class** (for collapsing) |
| --- | --- | --- |
| *Narcan** | *Opioids (Overdose reversal)* | *Naloxone* |
| *Naloxone** | *Opioids (Overdose reversal)* | *Naloxone* |
| *Naltrexone** | *Opioids (MOUD)* | *Naltrexone* |
| *Vivitrol** |  | *Naltrexone* |
| *Iboga, Ibogaine** |  | *Iboga* |
| Diphenhydramine | Antihistamines | Diphenhydramine |
| Benadryl | Antihistamines | Diphenhydramine |
| Ativan | Benzodiazepines | Benzodiazepine |
| Alprazolam | Benzodiazepines | Benzodiazepine |
| Benzo | Benzodiazepines | Benzodiazepine |
| Xanax | Benzodiazepines | Benzodiazepine |
| Valium | Benzodiazepines | Benzodiazepine |
| Benzodiazepine | Benzodiazepines | Benzodiazepine |
| Dextromethorphan | Cough Suppressants | Dextromethorphan |
| DXM | Cough Suppressants | Dextromethorphan |
| Vicodin | Opioids | Hydrocodone |
| Hydrocodone | Opioids | Hydrocodone |
| Oxycodone | Opioids | Oxycodone |
| Oxycontin | Opioids | Oxycodone |
| Fentanyl | Opioids | Fentanyl |
| Heroin* | Opioids | Heroin |
| Percocet | Opioids | Oxycodone |
| Opioid | Opioids | Opioid |
| Carfentanil | Opioids | Carfentanil |
| Methadone | Opioids (MOUD) | Methadone |
| Sublocade | Opioids (MOUD) | Buprenorphine |
| Buprenorphine | Opioids (MOUD) | Buprenorphine |
| Suboxone | Opioids (MOUD);  Opioid Antagonists (Naloxone) | Buprenorphine |
| Kratom | Opioids ** | Kratom |
| Adderal | Stimulants | Dextroamphetmine/Amphetamine |
| * Indicates medication names that were manually added to the analysis. | | |

1. Sample posts (paraphrased/partial/deidentified)

- “*The most [take-homes] one can get at [my opioid treatment program] is 1 week. I asked if they would have to make special exceptions because of the [COVID-19] crisis. I was told that nobody has to do anything for us... I understand that take home medications could kill kids, pets, etc[.] if someone is irresponsible[,] but I think they should let up a little bit.”.*
- *‘… I usually have 2 week [take-homes], I'm getting 28 days now [because] of COVID19. My clinic has ONLY been testing unstable patients, new patients, or ones with recently failed [urinalyses]. But all of it is up to the individual clinics or corporate owners of the clinic.’*
- *‘… What has helped you stay clean? [Person] is trying to stay clean now from heroin and opiates. He won't go to rehab and getting clean himself and I am trying to support him best I can. He says doing stuff helps to keep his mind busy, but it has been hard with COVID and we have a [young child].’*
- *‘Hello, I'm on methadone at ... I've been there … [years]. NO dirty urines. And [I am] compliant. However[,] with my age … and my health issues are [chronic conditions]. Plus[,] I had cancer … [years] ago. Which means my immune system is comprised. All documented via paperwork from doctors/emergency rooms plus my medication lists… I've not been given the take-home doses nor have others that have health issues causing us to be high risks [] for [COVID-19]. What can we do? I've talked to the drug board, and health department. As have others. We feel very afraid that we will die trying to get dosed. It's maddening knowing the clinic has permission to give take homes but refuse. They just "blow us off" saying they will call etc[.] etc. But no one of us has been called yet.’*
- *‘I live in … My clinic is currently giving me take homes as they are with whoever already had take homes and clean urines. … [someone] I work with just tested positive and a [colleague from the location] I work at just passed from COVID. So[,] the rest of us just got the order to [self-quarantine] for 14 days. Problem is nobody but the nurses and security [are] at the program. No counselors[,] no doctors[,] nothing. So how do you recommend I go about telling them this? ... I just want to keep my fellow patients worry free (from catching anything from me, GOD FORBID if I have it)"’*
- *‘I am indeed. I'm currently at …mg. Down from [previous higher dosage]. My clinic won't allow tapering while on [COVID] take homes.*

*I test positive for [THC] so I've never been allowed take homes until [COVID] happened. Then they allowed me 2 weeks at a time until june [date] and then back to normal daily dosing… [I am] so ready to be off. 2 years of methadone is long enough for me.’*

- *“Any suggestions on what's helped someone stay clean or how to support a loved one are appreciated."*
